# Augmenting CO_2_ Absorption Flux through a Gas–Liquid Membrane Module by Inserting Carbon-Fiber Spacers

**DOI:** 10.3390/membranes10110302

**Published:** 2020-10-22

**Authors:** Luke Chen, Chii-Dong Ho, Li-Yang Jen, Jun-Wei Lim, Yu-Han Chen

**Affiliations:** 1 Department of Water Resources and Environmental Engineering, Tamkang University, Tamsui, New Taipei 251, Taiwan; luke@mail.tku.edu.tw; 2 Department of Chemical and Materials Engineering, Tamkang University, Tamsui, New Taipei 251, Taiwan; bbb67890g1@gmail.com (L.-Y.J.); cyh84505@gmail.com (Y.-H.C.); 3 Department of Fundamental and Applied Sciences, HICoE-Centre for Biofuel and Biochemical Research, Institute of Self-Sustainable Building, Universiti Teknologi PETRONAS, Seri Iskandar, Perak Darul Ridzuan 32610, Malaysia

**Keywords:** carbon dioxide absorption, MEA solvent, mass transfer, Sherwood number, membrane contactor, concentration polarization

## Abstract

We investigated the insertion of eddy promoters into a parallel-plate gas–liquid polytetrafluoroethylene (PTFE) membrane contactor to effectively enhance carbon dioxide absorption through aqueous amine solutions (monoethanolamide—MEA). In this study, a theoretical model was established and experimental work was performed to predict and to compare carbon dioxide absorption efficiency under concurrent- and countercurrent-flow operations for various MEA feed flow rates, inlet CO_2_ concentrations, and channel design conditions. A Sherwood number’s correlated expression was formulated, incorporating experimental data to estimate the mass transfer coefficient of the CO_2_ absorption in MEA flowing through a PTFE membrane. Theoretical predictions were calculated and validated through experimental data for the augmented CO_2_ absorption efficiency by inserting carbon-fiber spacers as an eddy promoter to reduce the concentration polarization effect. The study determined that a higher MEA feed rate, a lower feed CO_2_ concentration, and wider carbon-fiber spacers resulted in a higher CO_2_ absorption rate for concurrent- and countercurrent-flow operations. A maximum of 80% CO_2_ absorption efficiency enhancement was found in the device by inserting carbon-fiber spacers, as compared to that in the empty channel device. The overall CO_2_ absorption rate was higher for countercurrent operation than that for concurrent operation. We evaluated the effectiveness of power utilization in augmenting the CO_2_ absorption rate by inserting carbon-fiber spacers in the MEA feed channel and concluded that the higher the flow rate, the lower the power utilization’s effectiveness. Therefore, to increase the CO_2_ absorption flux, widening carbon-fiber spacers was determined to be more effective than increasing the MEA feed flow rate.

## 1. Introduction

Biogas contains more than just hydrocarbons (typically ranging between 35% and 75% vol). Through processing and conditioning, the purity of biogas is upgraded, which adds to its value. Upgrading is the process of the separation of methane from carbon dioxide and other gases from biogas. In the biogas upgrading process, impurities such as CO_2_ (30–45%) and H_2_S (0.5–1%) are removed down to a level that satisfies specifications for biogas production or pipeline transport. Flue gases from fossil fuel combustion contain CO_2_ that needs to be removed in order to reduce greenhouse gas emissions. Of all these applications, absorption (physical or chemical) is the most common purification technology for gas separation. Many past CO_2_ absorption studies have focused on solvent development, aiming to optimize solvent formulation and achieve the lowest possible energy requirement in an efficient, stable, and environmentally friendly process [1,2]. Membrane separation technology has been widely applied to gas absorption and metal ion removal because of its simplicity, its low consumption of energy, the required pressure, and the possibility of using low-grade energy sources [3]. Ramakula et al. [4] investigated the membrane technique that is used to separate radioactive metal ions in many separation processes such as liquid/liquid and gas/liquid systems. The mass transfer behaviors of membrane consisting of Knudsen diffusion, molecular diffusion, surface diffusion, and viscous flow, referred to as the dusty gas model, have been studied by many researchers, and Knudsen molecular diffusion transition models [5,6] have been widely and successfully applied to express mass flux [7,8]. The membrane system’s simple configuration is easy for continuous operations, modulation arrangement, and scale-up extrapolation. The application of membrane contactors to the CO_2_ absorption process allows soluble gas mixture components to be selectively absorbed during a chemical reaction on the membrane’s surface in its liquid phase [9,10,11]. The durability and reusability of some materials such as PMSQ aerogel and Al_2_O_3_/SiO_2_-FAS for CO_2_ absorption were proven by Lin et al. [12]. The membranes of hybrid silica aerogels and highly porous PVDF/siloxane nanofibrous have been combined to enhance CO_2_ absorption flux considerably [13].

Microporous hydrophobic membrane devices act as a gas absorber when gas flows on one side and diffuses through the membrane pores while the amine solution flows on the other side in direct contact with the membrane surface [14]. The application of a gas-absorption membrane contactor aims to overcome operational limitations such as conventional packed columns, which suffer from liquid channeling, flooding, entrainment, and foaming [15]. Rongwong et al. [16] provided a better demonstration of the simultaneous removal of CO_2_ and H_2_S in the gas-absorption operations of membrane than conventional gas-absorption processes. The separation efficiency depends on the distribution coefficient of gas solute in both gas and liquid phases [17]. The membrane absorption method associated with the advantages of chemical absorption and the membrane separation technique allows us to selectively absorb the soluble gas mixture components in the solvent on the membrane surface [18]. Moreover, the advantage of a higher specific area can compensate for the disadvantages in the membrane contactor of an additional mass transfer resistance and control pressure due to the membrane’s presence in the contacting phases [19].

Concentration polarization builds up concentration gradients in the absorbent stream, leading to a decreased mass transfer rate [20] due to the diffusion and reaction occurring at the membrane–liquid interface, and thus decreasing the mass flux [21]. The concentration polarization effect plays a vital role in reducing transmembrane mass flux in the flat sheet membrane contactor system, as reported in the most studied configuration of the direct contact membrane absorption system. Kim et al. [22] conducted an experimental work in which they created a lab-scale module of enhanced CO_2_ absorption flux by conditioning membrane materials of both hydrophobic (bulk) and hydrophilic (surface) properties in order to simultaneously avoid wetting the solution and fouling the enzymes. Various approaches have been proposed to reduce the concentration polarization effect using eddy promoters in spacer-filled channels [23].

A prospective strategy proposing an alternative [24] included breaking down the laminar sublayer in a turbulent boundary layer region to destroy the viscous laminar sublayer adjacent to the absorber plate, and adding carbon-fiber spacers into the flowing channel. Moreover, Ho et al. [25] pointed out that if an eddy is only created close to the membrane surface where the mass transfer takes place, it should not be overly disturbed and should avoid exceptional power consumption. Santos et al. [26] explored how absorption efficiency in a parallel-plate gas–liquid polytetrafluoroethylene (PTFE) membrane contactor was enhanced by inserting eddy promoters, as compared to those of empty channel devices. The current design’s advantage by inserting eddy promoters is evident and provides a remarkable opportunity for the absorption efficiency of parallel-plate gas–liquid PTFE membrane contactor to be improved by a maximum of 80%, and the turbulence enhancement effectively raised to a higher convective mass-transfer coefficient [27]. Theoretical and computational studies have also been conducted to model the system of the CO_2_ absorption analogized to the membrane distillation system except for the occurring reactions [28]. Testing was carried out mathematically and experimentally to identify the influences of the mass-transfer rate of CO_2_ absorption efficiency based on physical absorption [29]. Research and development efforts can be made in the membrane contacting field employing spacer-filled channels [30] to minimize the concentration polarization effect to achieve higher mass transfer rates.

The advantage of chemical absorption technology is that it has been commercialized for many decades with various and mixed amines [31], used widely to enhance CO_2_ capture efficiency and reduce the regeneration cost [32]. In the present work, we used a theoretical model and performed experimental work to investigate CO_2_ absorption into monoethanolamide (MEA) using a parallel-plate gas–liquid PTFE membrane contactor. The microporous hydrophobic membrane device acts as a gas absorber for which the gas flows on one side and diffuses through the membrane pores while the amine solution flows on the other side, directly in contact with the membrane surface. The mass-balance and chemical reaction equations were formulated to simulate the hydrophobic porous membrane contactor system [33]. The turbulent intensity induced by inserting carbon-fiber spacers in the MEA absorbent flow channel was examined. A one-dimensional steady-state theoretical model was developed to simulate a more efficient absorption module for CO_2_ by amine solutions as chemical absorbents under concurrent-flow and countercurrent-flow operations. The channel’s mass transfer enhancement factor with the insertion of carbon-fiber spacers was correlated with the experimental data. The trade-off between increasing CO_2_ permeates and the power utilization’s effectiveness was evaluated to find the trend of economic feasibility in channel designs and system operations.

## 2. Theoretical Model

The following assumptions regarding the CO_2_ absorbed by the MEA absorbent through a membrane system were made:

1. The system is operated under normal pressure conditions;
2. The membrane is a porous hydrophobic media and is not wetted by the liquid MEA;
3. The membrane material does not react with liquid MEA;
4. Henry’s law applies to the interface between the gas phase and the liquid phase.

**Figure 1:**
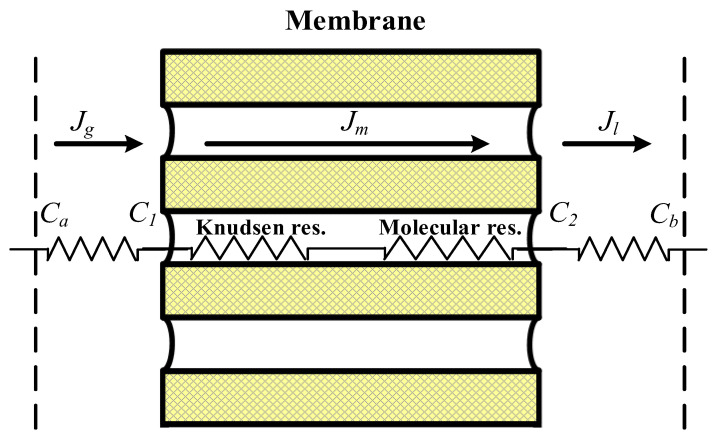
Schematic diagram of mass transfer resistances in a gas–liquid membrane contactor.

Three mass transfer resistances were built up across the membrane between the two bulk flows in series, as illustrated in Figure 1. The first resistance is the solute gas transfers into the membrane surface from the bulk gas flow. The second resistance is the transmembrane mass flux via Knudsen diffusion and molecular diffusion through the membrane pores. The third resistance is the gas solute that reaches the membrane–liquid interface and reacts with the MEA absorbent. The mass transfer across the concentration boundary layers to and from the membrane surfaces was determined through convective mass transfer coefficients and the dimensionless Henry’s law constant $H_{C}=C_{2}/C_{1}=0.73$ [33]. Since the reaction is quick, resistance is controlled by a convective mass transfer that depends on the boundary layer’s flow regime of the MEA liquid stream.

The mass transfer in the gas phase of CO_2_ is driven by the concentration gradient between gas bulk flow and membrane surface on the gas side as depicted below:

(1) $$J_{g}=k_{a}\left(C_{a\left(g\right)}-C_{1\left(g\right)}\right)$$

The mass transfer in the membrane is driven by the concentration gradient between both membrane surfaces. For mass transfer in the membrane, both Knudsen diffusion and molecular diffusion were considered [34]. The mass flux of CO_2_ can be evaluated using a membrane permeation coefficient ($c_{m}$) and the trans-membrane saturation partial pressure differences (ΔP) of CO_2_ [35]:

(2) $$J_{m}=c_{m}(P_{1}-P_{2})={c_{m}\frac{dP}{dC}|}_{C_{mean}}(C_{1}-C_{2(g)})=c_{m}RT(C_{1}-\frac{{K^{\prime }}_{ex}C_{2\left(\ell \right)}}{H_{C}})=K_{m}(C_{1}-\frac{{K^{\prime }}_{ex}C_{2\left(\ell \right)}}{H_{C}})$$

The equilibrium constant $K_{ex}={[\mathrm{MEACOO}}^{-}{][H}^{+}]/\{[CO_{2}][\mathrm{MEA}]\}=1.25\times 10^{-5}$ with the reduced equilibrium constant $K_{ex}^{\prime }=K_{ex}{[\mathrm{MEA}]/[H}^{+}]$ is specified at T=298 K [36] for CO_2_ absorbed in aqueous MEA absorbent. Based on the mean free path of the CO_2_ molecule and the membrane pore size, Knudsen diffusion and molecular diffusion [21,37] were considered to determine the membrane permeation coefficient:

(3) $$c_{m}={\left(\frac{1}{c_{K}}+\frac{1}{c_{M}}\right)}^{-1}={\left\{{\left[1.064\frac{\varepsilon r}{\tau \delta_{m}}{\left(\frac{M_{w}}{RT_{m}}\right)}^{1/2}\right]}^{-1}+{\left[\frac{1}{{\left|Y_{m}\right|}_{\ln}}\frac{D_{m}\varepsilon }{\delta_{m}\tau }\frac{M_{w}}{RT_{m}}\right]}^{-1}\right\}}^{-1}$$

where tortuosity (τ) can be estimated using the porosity of the membrane [38] as

(4) τ=1/ε

The CO_2_ mass transfer from the membrane surface to the liquid phase is driven by the CO_2_ concentration gradient between the membrane surface and the liquid bulk flow and is depicted as

(5) $$J_{\ell }=k_{b}\left(\frac{{K^{\prime }}_{ex}C_{2\left(\ell \right)}}{H_{C}}-\frac{C_{b\left(\ell \right)}}{H_{C}}\right)$$

By continuity, the mass fluxes from the gas feed side, transferring through the membrane and then being absorbed into the liquid feed side, are all equal in number as depicted below:

(6) $$J_{i}=J_{g}=J_{m}=J_{\ell }\;\;\;i=carbon,empty$$

**Figure 2:**
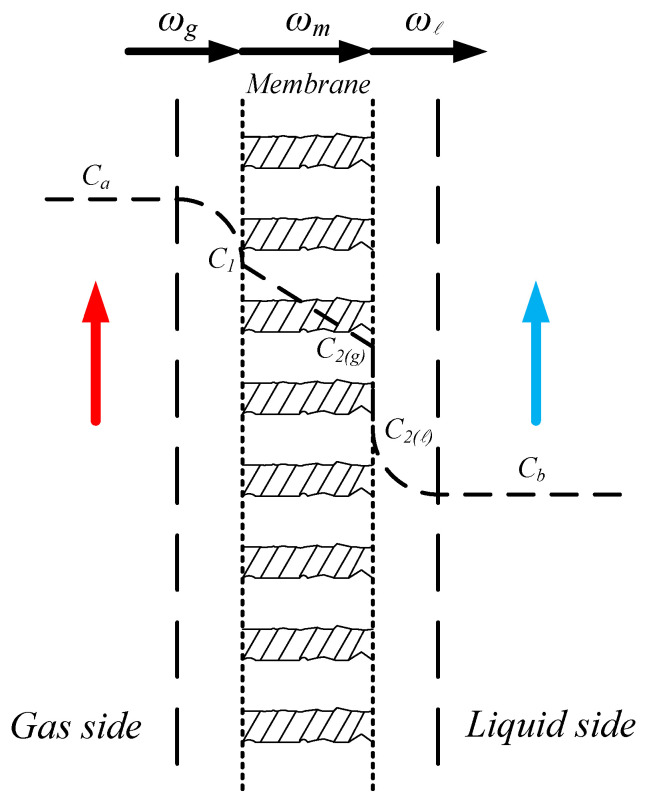
Schematic representation of the CO_2_ concentration variation from the gas phase to the liquid phase through the membrane.

The CO_2_ concentration variation from the gas phase to the liquid phase through the membrane is illustrated in Figure 2.

With the continuity of mass flux depicted in Equation (6), the CO_2_ concentrations in the buck flow of both gas and liquid streams and the CO_2_ concentrations on the membrane surfaces of both gas and liquid sides can be related by Equations (7) and (8).

(7) $$C_{a\left(g\right)}=C_{1\left(g\right)}+\frac{k_{m}}{k_{a}}\left(C_{1\left(g\right)}-\frac{{K^{\prime }}_{ex}C_{2\left(\ell \right)}}{H}\right)$$

(8) $$\frac{C_{b\left(\ell \right)}}{H}=\frac{{K^{\prime }}_{ex}C_{2\left(\ell \right)}}{H}-\frac{k_{m}}{k_{b}}\left(C_{1\left(g\right)}-\frac{{K^{\prime }}_{ex}C_{2\left(\ell \right)}}{H}\right)$$

Subtracting Equation (7) from Equation (8), one can obtain Equation (9) which can be used to derive a concentration polarization coefficient $\gamma_{m}$ as defined in Equation (10). The concentration polarization coefficient $\gamma_{m}$ was used to measure the dominance of mass transfer resistances in the CO_2_/MEA absorption system. A higher $\gamma_{m}$ value represents less mass transfer resistance. The concentration polarization is controlled by the boundary layers of both gas and liquid streams. To reduce the undesirable influence on the permeate flux, one needs to disrupt the boundary layers to increase the concentration polarization coefficient $\gamma_{m}$ to reduce the mass transfer resistances.

(9) $$C_{a\left(g\right)}-\frac{C_{b\left(\ell \right)}}{H}=\left(C_{1\left(g\right)}-\frac{{K^{\prime }}_{ex}C_{2\left(\ell \right)}}{H}\right)\left(1+\frac{k_{m}}{k_{a}}+\frac{k_{m}}{k_{b}}\right)$$

(10) $$\gamma_{m}=\frac{\left(C_{1\left(g\right)}-\frac{{K^{\prime }}_{ex}C_{2\left(\ell \right)}}{H}\right)}{\left(C_{a\left(g\right)}-\frac{C_{b\left(\ell \right)}}{H}\right)}=\frac{k_{a}k_{b}}{k_{a}k_{b}+k_{m}k_{a}+k_{m}k_{b}}$$

**Figure 3:**
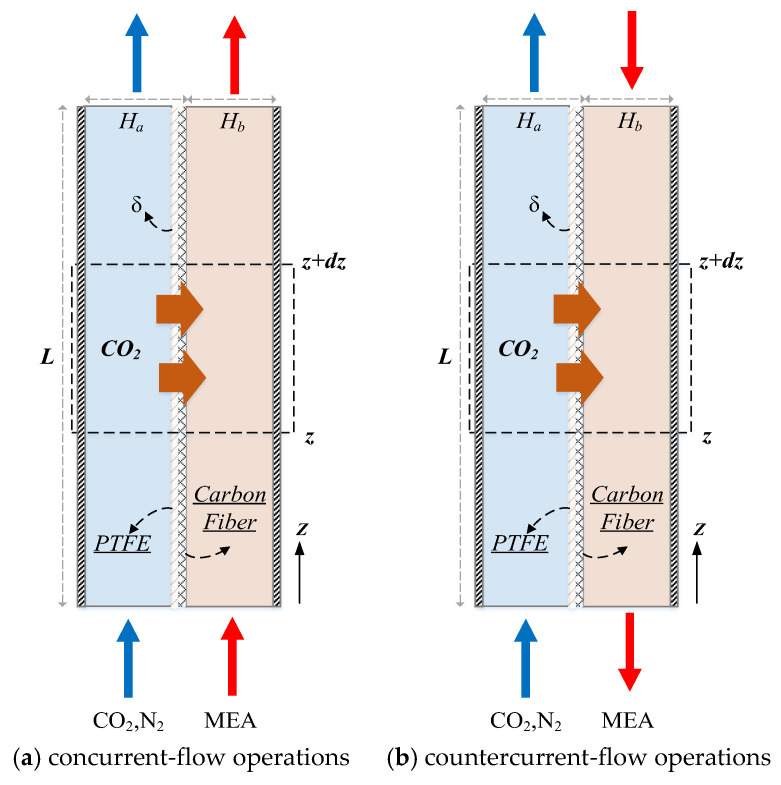
Schematic representation of CO_2_ absorption by monoethanolamide (MEA) for concurrent- and countercurrent-flow operations in parallel-plate membrane gas–liquid contactors. (**a**) concurrent-flow operations; (**b**) countercurrent-flow operations.

The CO_2_/MEA membrane absorption module configuration includes two parallel-plate flow channels separated by a membrane as a gas–liquid contactor. The system operates under both concurrent-flow and countercurrent-flow operations. The module has dimensions of length *L* and width *W*. The spaces between the membrane and the left or right plate ($H_{a}$ or $H_{b}$) are where the CO_2_ gas feed or MEA liquid absorbent flow through, respectively, as shown in Figure 3.

The mass balances of gas feed and liquid absorbent streams made within a finite system element respectively give:

(11) $$\frac{dC_{a}}{dz}=\frac{-W}{Q_{a}}\left[K_{m}\left(C_{1}-\frac{{K^{\prime }}_{ex}C_{2}{}_{\left(\ell \right)}}{H_{C}}\right)\right]=\frac{-W}{Q_{a}}\left[K_{m}\gamma_{m}\left(C_{a}-C_{b}\right)\right]$$

(12a) $$\begin{array}{ll} \frac{dC_{b}}{dz} & =\frac{WH(-k_{CO_{2}}C_{b})}{Q_{b}}+\frac{W}{Q_{b}}\left[K_{m}\left(C_{1}-\frac{{K^{\prime }}_{ex}C_{2}{}_{\left(\ell \right)}}{H_{C}}\right)\right]\\ & =\frac{WH(-k_{CO_{2}}C_{b})}{Q_{b}}+\frac{W}{Q_{b}}\left[K_{m}\gamma_{m}\left(C_{a}-C_{b}\right)\right]\\ & \text{for}\;\text{concurrent-flow}\;\text{operations} \end{array}$$

(12b) $$\begin{array}{ll} \frac{dC_{b}}{dz} & =\frac{(WH)k_{CO_{2}}C_{b}}{Q_{b}}-\frac{W}{Q_{b}}\left[K_{m}\left(C_{1}-\frac{{K^{\prime }}_{ex}C_{2}{}_{\left(\ell \right)}}{H_{C}}\right)\right]\\ & =\frac{WH(k_{CO_{2}}C_{b})}{Q_{b}}-\frac{W}{Q_{b}}\left[K_{m}\gamma_{m}\left(C_{a}-C_{b}\right)\right]\\ & \text{for}\;\text{concurrent-flow}\;\text{operations} \end{array}$$

where *z* is the coordinate along with the fluid flowing direction, and Equations (11), (12a) and (12b) are the mass balances for the gas and liquid phases of CO_2_ in MEA absorbent under concurrent-flow and countercurrent-flow operations. The CO_2_ concentrations in the gas feed stream, MEA liquid stream, and membrane surfaces along the module’s length were solved using the fourth-order Runge–Kutta method to determine the convective mass transfer coefficient. Hence, the CO_2_ absorption flux was obtained.

For the membrane contactor, using empty channels under laminar flow, the commonly used correlation [39] is:

(13) $$Sh_{lam}=0.023\;Re^{0.8}Sc^{0.33}$$

The extent of mass-transfer rate enhancement is frequently expressed by an enhancement factor, which is the ratio of the improved channel’s mass transfer coefficient to that of the empty channel. Similarly, the enhancement factor for the mass transfer coefficient can be defined for membrane gas–liquid contactors using the insertion of carbon-fiber spacers instead of the empty channel as follows:

(14) $$Sh^{E}=\frac{k_{b}d_{h},{}_{carbon}}{D_{b}}=\alpha^{E}Sh_{lam}$$

**Figure 4:**
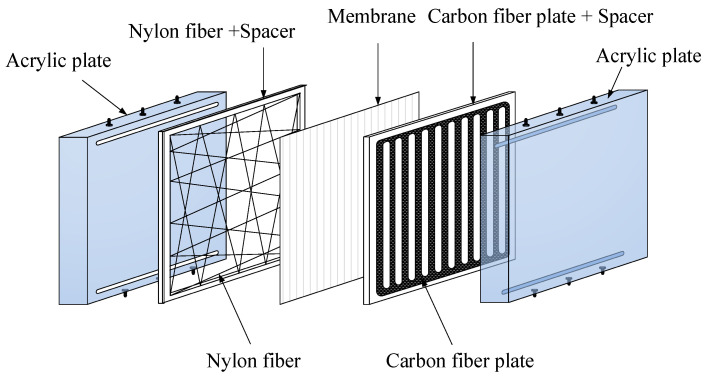
Components of a gas–liquid membrane contactor for the empty channel and the channel with the inserted carbon-fiber spacers.

The Sherwood number of inserted carbon-fiber spacers can be incorporated into four dimensionless groups using Buckingham’s π theorem:

(15) $$Sh^{E}=f(\frac{d_{h,carbon}}{d_{h,empty}},Re,Sc)$$

where $d_{h,carbon}$ is the equivalent diameter of inserted carbon-fiber spacers while $d_{h,empty}$ is the hydraulic diameter of the empty channel, as shown in Figure 4.

The expenses linked to the increase in power consumption are inevitable because the device was inserted with carbon-fiber spacers into the MEA flowing channel as eddy promoters. Power consumption due to the friction losses of a gas–liquid membrane contactor, which includes the contributions from the gas side and the MEA side, can be determined using the Fanning friction factor $f_{F}$ [40]:

(16) $$H_{i}=Q_{a}\;\rho_{CO{\;}_{2}}\;\ell w_{f,CO{\;}_{2}}+Q_{b}\;\rho_{MEA}\ell w_{f,MEA}\;\;\;i=carbon,empty$$

(17) $$\ell w_{f,j}=\frac{2f_{F,j}{\bar{v}}_{j}^{2}L}{d_{h,i}},\;j=CO_{2},MEA$$

The average velocity and equivalent hydraulic diameter of each flow channel are estimated as follows:

(18) $${\bar{\nu }}_{CO_{2}}=\frac{q_{a}}{\left(HW\right)}\;,\;{\bar{\nu }}_{MEA}=\frac{q_{b}}{\left(HW-D_{1}W_{1}N_{1}\right)}$$

(19) $$d_{h,CO_{2}}=\frac{4\left(HW\right)}{2(H+W)},\;d_{h,MEA}=\frac{4\left(HW-D_{1}W_{1}N_{1}\right)}{2\left(H+W+D_{1}N_{1}\right)}$$

The Fanning friction factor can be estimated using a correlation based on the channel’s aspect ratio (β=H/W) [41]:

(20) fF=24(1−1.3553β+1.9467β 2−1.7012β 3+0.9564β 4−0.2537β 5)/Re

The relative extents $I_{E}$ and $I_{P}$ of mass flux enhancement and power consumption increment, respectively, were illustrated by calculating the percentage increase in the device with the inserted carbon-fiber spacers, based on the device of the empty channel:

(21) $$I_{E}=\frac{J_{carbon}-J_{empty}}{J_{empty}}\times 100\%$$

(22) $$I_{P}=\frac{H_{carbon}-H_{empty}}{H_{empty}}\times 100\%$$

where the subscripts “carbon” and “empty” represent the channels where carbon-fiber spacers were inserted and the empty channel.

## 3. Experimental Study

**Figure 5:**
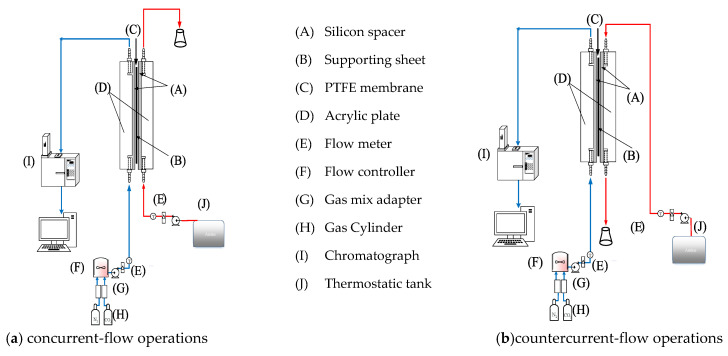
Experimental setup for parallel-plate membrane gas–liquid contactors. (**a**) concurrent-flow operations; (**b**) countercurrent-flow operations.

**Figure 6:**
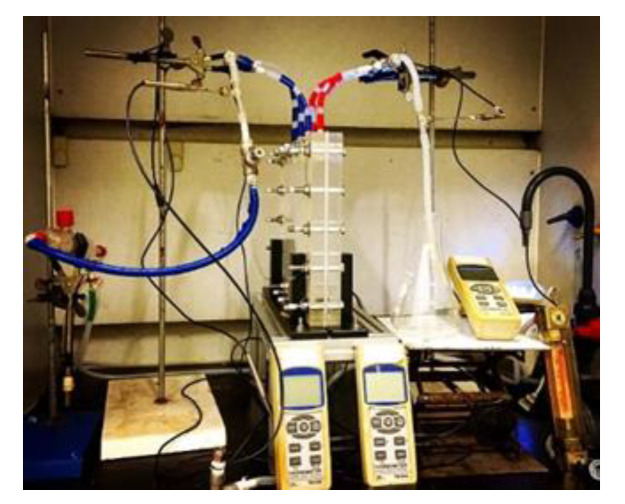
A photo of a real experimental setup.

A schematic diagram of the experimental setup of the parallel-plate gas–liquid membrane contactor for CO_2_ absorption by an MEA absorbent is assembled, as illustrated in Figure 5. A photo of the real experimental setup is shown in Figure 6. With acrylic plates used as the outside walls, the module contains two types of flow channels: the empty channel and the channel where carbon-fiber spacers were inserted into the MEA feed flow. The empty channel is constructed with a 0.2 mm nylon fiber-routed supporting sheet. The carbon-fiber spacer is constructed with a 1 mm-thick carbon-fiber sheet with open slots among a parallel carbon-fiber strip of 2 or 3 mm width placed on the liquid MEA side of the membrane acting as eddy promoters.

A gas mixture containing CO_2_ and N_2_ was introduced from the gas mixing tank to feed into one side of the membrane contactor. Aqueous amine (MEA) solution was chosen as the liquid absorbent flowing through the other side of the membrane from a reservoir. Two parallel-plate flow sub-channels (*L* = 0.21m, *W* = 0.29m, *H* = 0.02m) separated with a gas–liquid membrane contactor made of hydrophobic polytetrafluoroethylene (PTFE) membrane (ADVANTEC, Tokyo, Japan) were conducted as the experimental setup. The hydrophobic polytetrafluoroethylene (PTFE) membrane (ADVANTEC) with a nominal pore size of 0.1, porosity of 0.72, and thickness of 130 µm was used. The experimental runs were carried out for various MEA feed flow rates within the range of 5~10 cm^3^/s, while the gas flow rate was controlled at 5 cm^3/^s with two inlet CO_2_ concentrations of 30% and 40%, respectively. The CO_2_ concentration in the gas outlet stream was measured using gas chromatography (Model HY 3000 Chromatograph, China Corporation, New Taipei, Taiwan). Some experiments were regulated to control an appropriate pressure gradient to avoid bubbling for both modules—that of the empty channel and that of the channel with the inserted carbon-fiber spacers.

The experimental measurements of absorption efficiency, ω were defined as

(23) $$\omega (\%)=\left(\frac{C_{in}-C_{out}}{C_{in}}\right)\times 100$$

The precision index of experimental uncertainty of each individual measurement of $S_{\omega_{i}}$ is calculated as described by Moffat [42] directly from the experimental runs as follows:

(24) $$S_{\omega_{i}}={\left\{\sum_{i=1}^{N_{\exp}}\frac{{\left(\omega_{\exp}-\omega_{cal}\right)}^{2}}{N_{\exp}-1}\right\}}^{1/2}$$

and the uncertainty of the reproducibility of molar fluxes is associated with the mean precision index

(25) $$S_{{\bar{\omega }}_{i}}=\frac{S_{\omega_{i}}}{\sqrt{N_{\exp}}}$$

The mean precision index of the experimental measurements of absorption efficiency evaluated for concurrent-flow and countercurrent-flow operations is $8.82\times 10^{-3}\leq S_{{\bar{\omega }}_{i}}\leq 1.20\times 10^{-2}$.

The absorption flux’s experimental results prove the theoretical predictions’ validity by defining the accuracy [42] between the numerical solutions and the experimental results as follows:

(26) $$E(\%)=\frac{1}{N_{\exp}}\sum_{i=1}^{N_{\exp}}\frac{\left|\omega_{cal}-\omega_{\exp}\right|}{\omega_{\exp}}\times 100$$

where $\omega_{\exp}$ indicates the theoretical prediction of $\omega_{cal}$, while *N_exp_* and $\omega_{\exp}$ are the number of experimental measurements and the experimental data of $\omega_{cal}$. The error analysis of the experimental measurements determined by Equation (26) for both analytical models is 3.21≤E≤5.81.

## 4. Numerical Study

**Figure 7:**
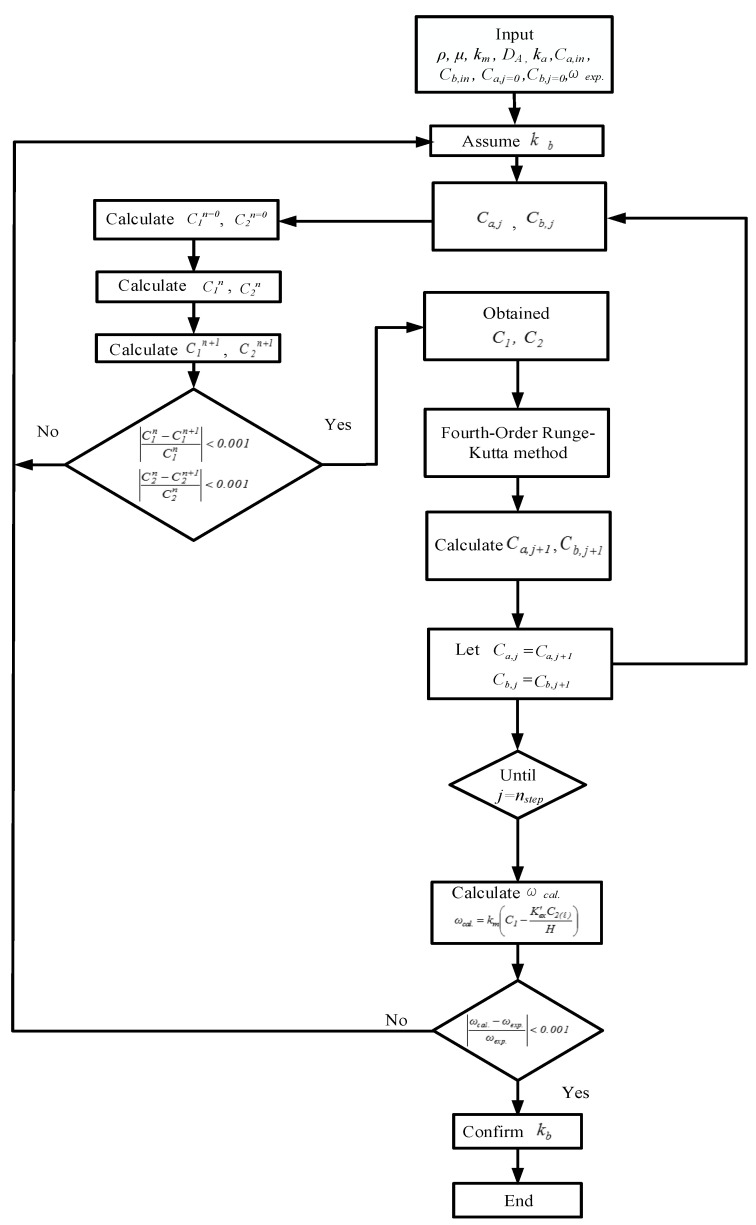
Calculation flow chart for determining CO_2_ concentrations in gas and liquid phases under concurrent-flow operations.

**Figure 8:**
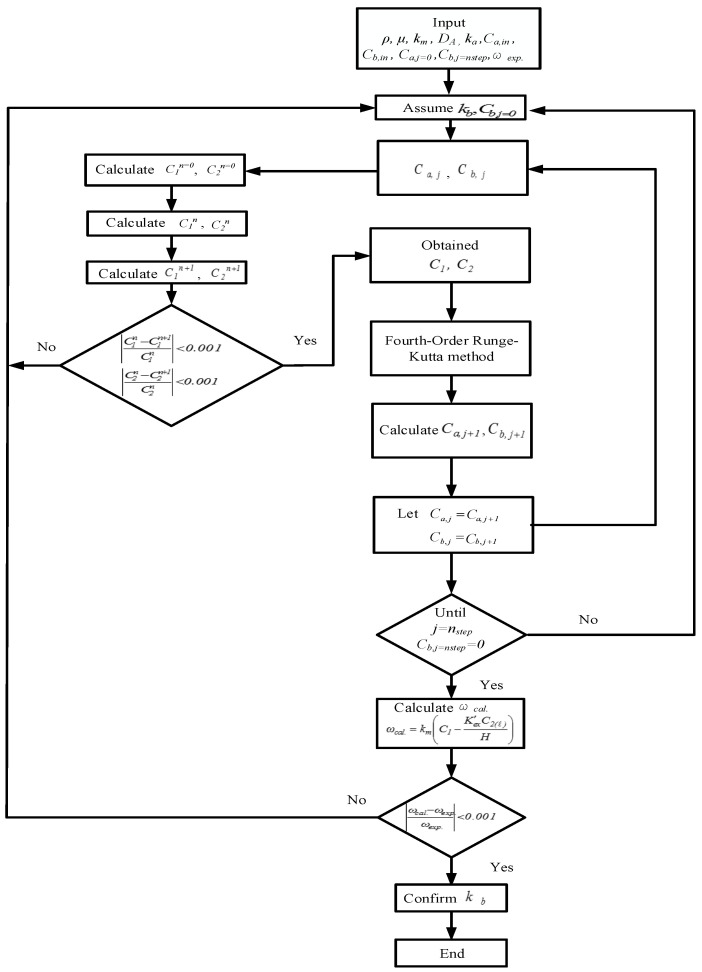
Calculation flow chart for determining CO_2_ concentrations in gas and liquid phases under countercurrent-flow operations.

An iterative procedure, as illustrated in Figure 7, was used to calculate the CO_2_ absorption flux $\omega_{cal}$ for concurrent-flow operation. The calculated CO_2_ absorption flux was $\omega_{cal}$, then compared with experimental CO_2_ absorption flux $\omega_{\exp}$ to check the convergence of the initial guess of the convective mass transfer coefficients *K_b_* in the liquid phase. Obtaining the convective mass transfer coefficients *K_b_*, one may apply the Range–Kutta scheme to solve Equations (11), (12a), and (12b) to obtain the CO_2_ concentration distribution not only in the gas/liquid buck flows but also on the membrane surfaces of both the gas and liquid sides under concurrent- and countercurrent-flow operations, respectively. In addition to the initial guess of the convective mass transfer coefficients *K_b_* in the liquid phase for concurrent-flow operation calculation, an additional guess of CO_2_ concentration at the inlet of MEA feed C_b,j=n_ needs to be specified as zero for countercurrent-flow operation calculation, as illustrated in Figure 8. Both experimental CO_2_ mass flux $\omega_{\exp}$ and CO_2_ concentration at the inlet of MEA feed C_b,j=n_ = 0 were used to check the convergence of the iterative calculation via the shooting method for the calculation of countercurrent-flow operation. When the iterative calculation is converged, the CO_2_ concentrations on the membrane surfaces and mass transfer coefficients *K_b_* were obtained, and the theoretical CO_2_ mass flux and CO_2_ absorption efficiency were then also obtained. Comparisons were made between the CO_2_ absorption efficiency of the channel where carbon-fiber spacers were inserted and that of the empty channel under both concurrent- and countercurrent-flow operations.

**Figure 9:**
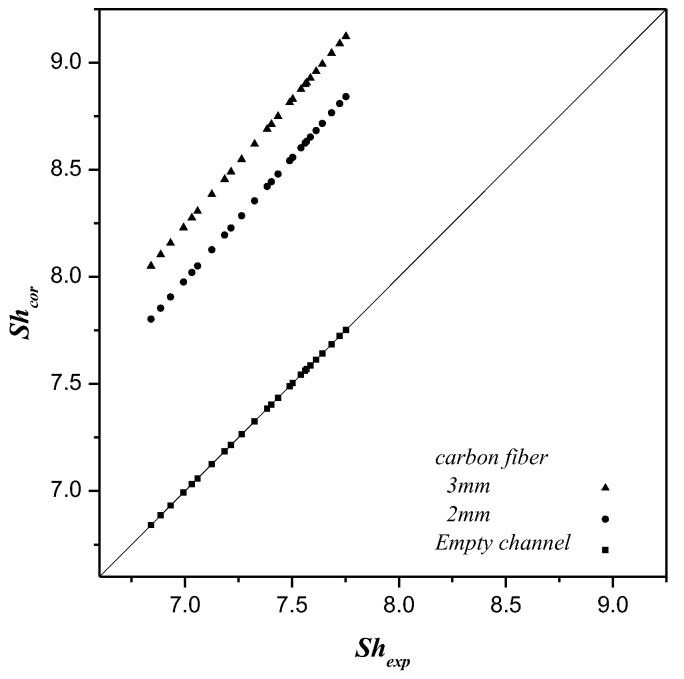
Comparison of calculated and experimental Sherwood numbers for the empty channel and the channels with different widths of carbon-fiber spacers.

The mass transfer coefficients expressed by the Sherwood number and determined by the theoretical model were used to compare and correlate with the experimental data, as shown in Figure 9. The enhanced factor $\alpha^{E}$ derived from the correlation of the Sherwood number for the channel with the inserted carbon-fiber spacers is determined via a regression analysis below:

(27) $$\alpha^{E}=0.008\ln{\left(\frac{d_{h,carbon}}{d_{h,empty}}\right)}^{2.334}$$

The correlated Sherwood numbers for the empty channel are in linear relation with the experimental data, as shown in Figure 9. The results validate that the correlation is also applicable to the channel where carbon-fiber spacers of 2 mm and 3 mm widths were inserted. The correlated Sherwood numbers, as shown in Figure 9, indicate that the mass transfer of the channel with 3 mm width carbon-fiber spacers inserted has a higher mass transfer than that of the channel with 2 mm width, and the mass transfer in the channels with the inserted carbon-fiber spacers is higher than that of the empty channel. Inserting carbon-fiber spacers disrupts the boundary layer on the membrane surface that reduces mass transfer resistance; hence, the permeate flux was enhanced. The larger the width of the carbon-fiber spacers inserted, the higher the turbulence intensity produced that results in a higher mass transfer or CO_2_ absorption flux.

## 5. Results and Discussions

### 5.1. Concentration Polarization

**Figure 10:**
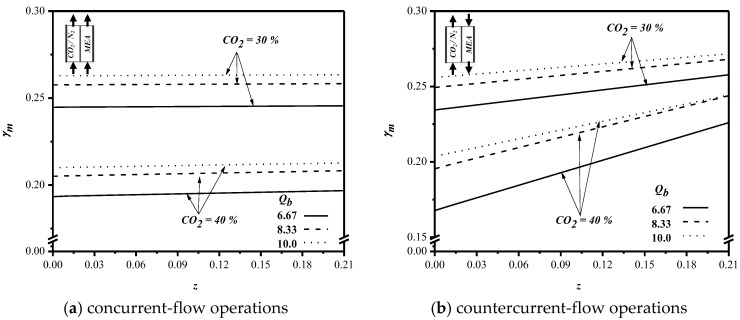
Effects of the MEA flow rate and feed CO_2_ concentration on $\gamma_{m}$. (**a**) concurrent-flow operations; (**b**) countercurrent-flow operations.

The concentration polarization dominates the mass transfer resistances on the boundary layers for both the gas and liquid streams, especially on the liquid side. With the predicted CO_2_ concentration distributions, the concentration polarization coefficients ($\gamma_{m}$) along the channel direction for various MEA feed flow rates and feed CO_2_ concentrations can be determined, as indicated in Figure 10. The higher the $\gamma_{m}$ value, the smaller the mass-transfer resistance. According to the concentration polarization coefficients ($\gamma_{m}$) shown in Figure 10, we found a higher $\gamma_{m}$ value resulting from the higher feed flow rate. The $\gamma_{m}$ value increases when MEA feed flow rates increase but decreases in the reverse flow direction of the MEA inlet feed owing to the concentration gradient decreasing between gas and liquid sides, since a higher feed flow rate can create a higher turbulent intensity on the boundary layer of the membrane surface to disrupt the occurrence of concentration polarization. In evaluating the effect of feed CO_2_ concentration on concentration polarization, we found a higher $\gamma_{m}$ value for 30% feed CO_2_ concentration when comparing with the $\gamma_{m}$ of 40% feed CO_2_ concentration. The higher the feed CO_2_ inlet concentration was, the bigger the CO_2_ concentration accumulated on the membrane surface was anticipated, and hence, lower concentration polarization coefficients ($\gamma_{m}$) or a larger mass transfer resistance were found, as shown in Figure 10. A similar effect of the feed flow rate and feed CO_2_ concentrations on concentration polarization coefficients $\gamma_{m}$ were found in concurrent- and countercurrent-flow operations.

**Figure 11:**
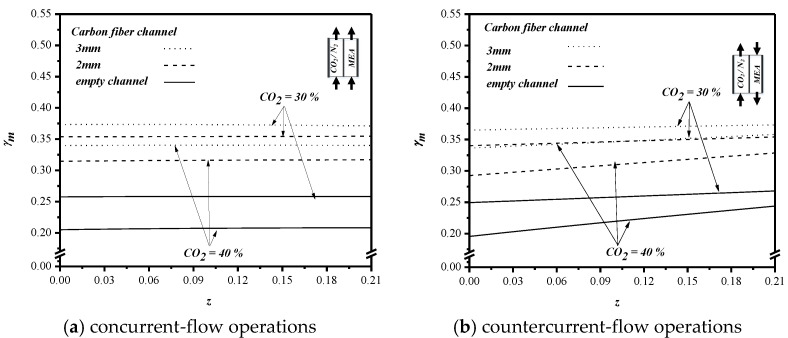
Effects of the width of carbon-fiber spacers and CO_2_ concentration on $\gamma_{m}$. (**a**) concurrent-flow operations; (**b**) countercurrent-flow operations.

The concentration polarization coefficient $\gamma_{m}$ values of the channels with the inserted carbon-fiber spacers of 3 and 2 mm width for feed CO_2_ concentration of 30% and 40% were compared to those of the device with the empty channel for both concurrent- and countercurrent-flow operations, as shown in Figure 11 for the same MEA feed flow rate *Q_b_* = $8.33\times 10^{-6}$ m^3^/s. The higher feed CO_2_ concentration causes a larger concentration polarization on the membrane surface, as explained before. When comparing the concentration polarization coefficient $\gamma_{m}$ value for the devices with the inserted carbon-fiber spacers of 3 or 2 mm width and those of the empty channel, we found that the $\gamma_{m}$ values are in descending order from 3 to 2 mm, and then the empty channel. The comparison concludes that the wider the carbon-fiber spacer the larger the turbulence intensity, hence resulting in a smaller mass transfer resistance. In other words, the turbulence induced by the wilder carbon-fiber spacers on the membrane surface results in a more convective mass transfer to disrupt concentration polarization that leads to a higher $\gamma_{m}$ value or a higher mass transfer rate.

### 5.2. CO_2_ Absorption Flux Enhancement

**Figure 12:**
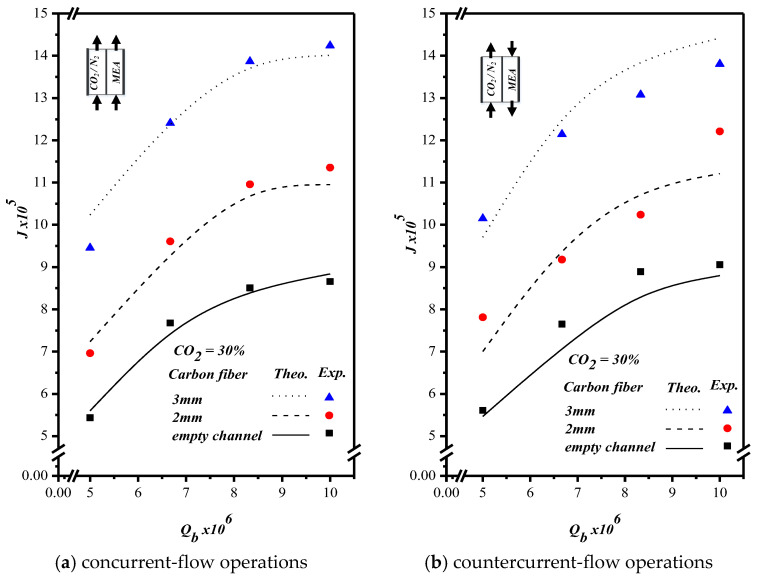
Effects of the MEA flow rate and carbon-fiber spacers’ width on CO_2_ absorption flux. (**a**) concurrent-flow operations; (**b**) countercurrent-flow operations.

In addition to investigating the effects of the feed flow rate and the width of carbon-fiber spacers on concentration polarization, this study also measured, predicted, and compared the effects on CO_2_ absorption flux for both concurrent- and countercurrent-flow operations, as depicted in Figure 12. As expected, both the increase of the MEA feed flow rate and that of the width of the carbon-fiber spacers results in more permeate flux. The measured CO_2_ absorption rate, concentration polarization coefficient $\gamma_{m}$ for various MEA feed flow rates, and feed CO_2_ concentration under concurrent- and countercurrent-flow operations are summarized in Table 1 and Table 2, respectively. A relative increase of permeate flux $I_{E}$ was used to compare the permeate flux of the channel with carbon-fiber spacers to that of the empty channel. The comparison showed that the increased $I_{E}$ ranged from 19.6% to 34.7% and from 49.7% to 80.0% for the channel with carbon-fiber spacers of 2 and 3 mm widths, respectively. In general, the CO_2_ absorption rate enhanced by the insertion of carbon-fiber spacers is more significant in countercurrent-flow operations than in concurrent-flow operations.

In fact, the higher deviation occurred at the lower feed flow rate due to the insensitivity of the flowmeter regulation, especially for $Q_{b}=6.67\times 10^{-6\;}m^{3}s^{-1}$. However, the theoretical predictions’ validity for both operations is in the good agreement with the experimental results confirmed by the precision index of experimental uncertainty of each individual measurement.

### 5.3. Energy Consumption

**Figure 13:**
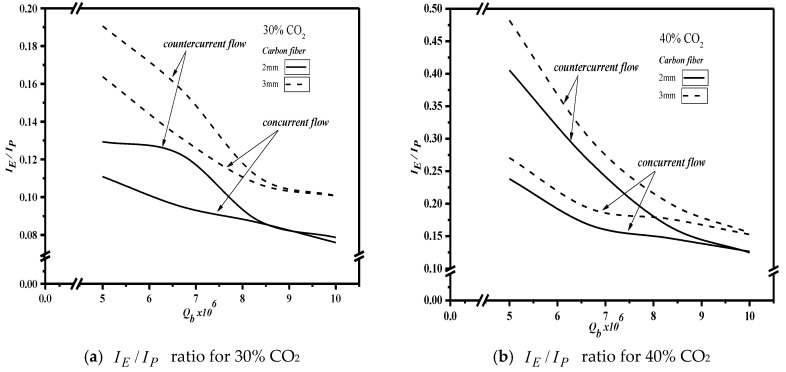
Effects of feed flow rate, spacers’ width, and feed CO_2_ concentration on $I_{E}/I_{P}$. (**a**) $I_{E}/I_{P}$ ratio for 30% CO_2_; (**b**) $I_{E}/I_{P}$ ratio for 40% CO_2._

Concerning the flow resistance caused by the insertion of carbon-fiber in the MEA channel, which consumes more power, this study further evaluated the channel design’s effectiveness by comparing the ratio of increment of CO_2_ absorption flux to the increment of power consumption, $I_{E}/I_{P}$. The effect of the feed flow rate, the width of carbon-fiber spacers, feed CO_2_ concentration, and concurrent-/countercurrent-flow operations on $I_{E}/I_{P}$ are summarized in Figure 13. The turbulence promotor through the insertion of carbon-fiber spacers in both concurrent- and countercurrent-flow operations aimed to achieve the augmented turbulence intensity in shrinking concentration polarization layers and enlarge the mass transfer coefficient as well, and thus, the absorption flux was enhanced. The reason behind the difference is that the countercurrent-flow operations run with a larger concentration gradient between gas and liquid than the concurrent-flow operations do. A higher feed CO_2_ concentration gives a higher $I_{E}/I_{P}$ ratio. The increase of the MEA feed flow rate gives a lower value of $I_{E}/I_{P}$, which implies that increasing the CO_2_ absorption flux with the expense of power by increasing the MEA feed rate is a less effective approach than changing carbon-fiber spacer widths. Comparing the effectiveness of the concurrent- and countercurrent-flow operation, we found that the $I_{E}/I_{P}$ values of the countercurrent-flow operation are all higher than those of the concurrent-flow operation. The comparison reveals that the countercurrent-flow operation can utilize power to increase the CO_2_ absorption flux more effectively than the concurrent-flow operation can. The increase of CO_2_ concentration gives a higher value of $I_{E}/I_{P}$, which reflects that this expense of energy consumption is more effective in increasing the absorption flux. In other words, the percentage of enhancement of the absorption flux is higher than that of the increment of energy consumption. In fact, consideration of the use of carbon-fiber spacers as turbulence promotors when making economic analyses creates two conflicting effects: the desirable improvement of the absorption flux and the undesirable increment of power consumption. The power consumption increment is relatively higher at a higher MEA feed flow rate with an essentially large turbulence resulting in a higher mass transfer coefficient for the device where carbon-fiber spacers are inserted. However, the higher absorption flux, in terms of $I_{E}/I_{P}$, indicates that a larger power consumption increment cannot create more absorption flux due to the relative rate of CO_2_ consumed by the limited equilibrium constant of the chemical reaction in the liquid side. Therefore, the improvement of absorption flux using the device with the inserted carbon-fiber spacers can compensate for the increment of power consumption more effectively than increasing the MEA feed flow rate would.

As the $I_{E}/I_{P}$ values decrease when the MEA feed flow rate increases, one may notice that a comparatively smaller change of $I_{E}/I_{P}$ values was observed, as the feed flow rate is over $8.33\times 10^{-6}$ m^3^/s for both the carbon-fiber spacer with a width of 3 mm and that with a width of 2 mm. The same observation was also found for both concurrent- and countercurrent-flow operations. Note that the $I_{E}/I_{P}$ ratio of the channel with carbon-fiber spacers of a 3 mm width is higher than that of the channel with a 2 mm width. The comparison also confirms that to increase the CO_2_ absorption flux, widening carbon-fiber spacers is more effective than increasing the MEA feed flow rate. Therefore, comparisons between the 2 mm and 3 mm carbon-fiber spacers were made, considering the effective utilization of power consumption relative to the increase of the CO_2_ absorption flux to indicate the trend of economic feasibility when inserting a wider carbon-fiber spacer for the specific MEA feed rates used in this study.

## 6. Conclusions

A parallel-plate gas–liquid PTFE membrane contactor where carbon-fiber spacers were inserted to be used as eddy promoters to enhance the CO_2_ absorption by MEA was investigated. The theoretical predictions of the enhancement of CO_2_ absorption by inserting carbon-fiber spacers were calculated and validated using experimental data, and the correlated expression of the Sherwood number was obtained. Thorough comparisons of the CO_2_ absorption efficiency for various MEA feed flow rates, CO_2_ feed concentrations, and carbon-fiber spacer widths under concurrent- and countercurrent-flow operations were completed. The comparisons helped us to draw the following conclusions:

1. The higher the MEA feed rate, the lower the feed CO_2_ concentration, and wider carbon-fiber spacers result in a larger CO_2_ absorption rate for concurrent- and countercurrent-flow operations. A maximum of 80% enhancement in CO_2_ absorption efficiency was found in the device where carbon-fiber spacers were inserted compared to that in the empty channel device.
2. The CO_2_ absorption rate is higher for countercurrent operation than that for concurrent operation. The CO_2_ absorption flux is mainly driven by the overall CO_2_ concentration gradient along the channel direction. The overall CO_2_ concentration gradient for countercurrent operation is higher than that for concurrent operation of the system.
3. The ratio of increment of the CO_2_ absorption flux to the increment of power consumption was used to evaluate the power utilization’s effectiveness in augmenting the CO_2_ absorption rate in this system. The evaluation concluded that the power utilization is more effective for the channel where carbon-fiber spacers of 3mm were inserted than that of 2mm, and the higher the feed MEA flow rate, the lower the effectiveness of the power utilization. To increase the CO_2_ absorption flux, widening the carbon-fiber spacers is more effective than increasing the MEA feed flow rate.

A new device in this study includes the desirable effect of raising turbulence intensity as an alternative strategy [28] to the CO_2_ absorption in MEA through the membrane contactor. Though the mass transfer mechanism in this gas–liquid membrane contactor could be analogized from that of the previous work [28], the manners of transport through the membrane module are somewhat different, including the chemical reaction. In this paper, only the CO_2_ absorption efficiency and power utilization effectiveness were evaluated by inserting carbon-fiber spacers as an eddy promoter in the MEA feed channel. The alternative absorbent, membrane material, and module design require further investigation.

## Figures and Tables

**Table 1:** Effects of operating condition and spacers’ width on $\gamma_{m}$ for concurrent-flow operations.

$C_{in}$ (%)	$Q_{b}\times 10^{6}$ (m^3^ s^−1^)	Empty Channel		Inserted Carbon-Fiber Spacers							
				2 mm				3 mm			
		$J_{theo}\times 10^{5}$ (mol m^−2^ s^−1^)	$\gamma_{m}$	$J_{theo}\times 10^{5}$ (mol m^−2^ s^−1^)	E (%)	$\gamma_{m}$	$I_{E}$	$J_{theo}\times 10^{5}$ (mol m^−2^ s^−1^)	E (%)	$\gamma_{m}$	$I_{E}$
30	6.67	7.42	0.2450	9.27	3.52	0.3500	24.9	12.4	0.29	0.3709	67.1
	8.33	8.39	0.2578	10.7	2.55	0.3527	27.5	13.7	1.14	0.3725	63.3
	10.0	8.84	0.2630	11.0	3.55	0.3566	24.4	14.0	1.60	0.3749	58.4
40	6.67	8.75	0.1946	10.5	4.02	0.3122	20.0	13.1	4.04	0.3482	49.7
	8.33	9.45	0.2062	11.4	0.34	0.3153	20.6	14.3	0.17	0.3501	51.3
	10.0	9.79	0.2109	12.2	0.80	0.3189	24.6	15.0	2.26	0.3522	53.2

$\gamma_{m}$ data are the average value of the parallel-plate gas–liquid membrane contactor module.

**Table 2:** Effects of operating condition and spacers’ width on $\gamma_{m}$ for countercurrent-flow operations.

$C_{in}$ (%)	$Q_{b}\times 10^{6}\;(m^{3}\;s^{-1})$	Empty Channel		Inserted Carbon-Fiber Spacers							
				2 mm				3 mm			
		$J_{theo}\times 10^{5}$ (mol m^−2^ s^−1^)	$\gamma_{m}$	$J_{theo}\times 10^{5}$ (mol m^−2^ s^−1^)	E (%)	$\gamma_{m}$	$I_{E}$	$J_{theo}\times 10^{5}$ (mol m^−2^ s^−1^)	E (%)	$\gamma_{m}$	$I_{E}$
30	6.67	7.11	0.2426	9.58	4.34	0.3407	34.7	12.8	5.32	0.3653	80.0
	8.33	8.45	0.2578	10.9	6.09	0.3451	29.0	14.0	6.67	0.3679	65.5
	10.0	8.80	0.2614	11.2	2.46	0.3493	27.3	14.4	4.43	0.3704	63.6
40	6.67	8.65	0.1882	10.9	12.3	0.2999	26.0	13.8	4.83	0.3408	59.5
	8.33	9.88	0.2124	11.9	0.97	0.3051	20.4	15.1	5.16	0.3439	52.8
	10.0	10.2	0.2162	12.2	1.84	0.3091	19.6	15.5	3.51	0.3463	52.0

$\gamma_{m}$ data are the average value of the parallel-plate gas–liquid membrane contactor module.

## Abbreviations

C	Concentration (mol m^−3^)
$C_{mean}$	Mean value of C (mol m^−3^)
$c_{k}$	Membrane coefficient based on the Knudsen diffusion model (${\mathrm{mol}\;m}^{-2}{\mathrm{Pa}}^{-1}s^{-1}$)
$c_{M}$	Membrane coefficient based on the molecular diffusion model (${\mathrm{mol}\;m}^{-2}{\mathrm{Pa}}^{-1}s^{-1}$)
$c_{m}$	Membrane permeation coefficient (${\mathrm{mol}\;m}^{-2}{\mathrm{Pa}}^{-1}s^{-1}$)
$D_{1}$	Width of the inserting carbon-fiber spacers (m)
$D_{b}$	Diffusion coefficient of CO_2_ in MEA (m^2^ s^−1^)
$D_{m}$	Diffusion coefficient of N_2_ and CO_2_ in the membrane (m^2^ s^−1^)
$d_{h,i}$	Equivalent hydraulic diameter of channel (m), i=carbon,empty
E	Deviation of experimental results from the theoretical predictions
$f_{F}$	Fanning friction factor
H	Channel height (m)
$H_{C}$	Dimensionless Henry’s constant
$H_{i}$	Hydraulic dissipate energy (J kg^−1^), i=carbon,empty
$I_{E}$	Mass flux enhancement, defined by Equation (21)
$I_{P}$	Power consumption relative index, defined by Equation (22)
$J_{i}$	Molar flux (mol m^−2^ s^−1^)
$k_{a}$	Mass transfer coefficient in the gas feed flow side (m s^−1^)
$k_{b}$	Mass transfer coefficient in the liquid absorbent flow side (m s^−1^)
$K_{ex}$	Equilibrium constant
${K^{\prime }}_{ex}$	Reduced equilibrium constant
$K_{m}$	Overall mass transfer coefficient of membrane (m s^−1^)
k_CO__2_	Mass transfer of carbon dioxide (${\mathrm{mol}\;m}^{-2}s^{-1}$)
$\ell w_{f,j}$	Friction loss of CO_2_ (J kg^−1^), $j=CO_{2},MEA$
L	Channel length (m)
$M_{W}$	Molecular weight of water (kg mol^−1^)
N_exp_	Number of experimental measurements
$N_{1}$	Number of inserting carbon-fiber fins
$P_{1}$	Saturation vapor pressure in the gas feed flow side (Pa)
$P_{2}$	Saturation vapor pressure in the liquid absorbent flow side (Pa)
$Q_{a}$	Volumetric flow rate of the gas feed flow side (m^3^ s^−1^)
$Q_{b}$	Volumetric flow rate of the liquid absorbent flow side (m^3^ s^−1^)
R	Gas constant (8.314 J mol^−1^ K^−1^)
Re	Reynolds number
r	Membrane pore radius (m)
$S_{\omega }$	The precision index of an experimental measurements of molar flux (mol m^−2^ s^−1^)
$S_{{\bar{\omega }}_{i}}$	The mean value of $S_{\omega_{i}}$ (mol m^−2^ s^−1^)
Sc	Dimensionless Schmidt number
Sh^E^	Enhanced dimensionless Schmidt number
$Sh_{lam}$	Dimensionless Schmidt number for laminar flow
T	Temperature (°C)
$T_{m}$	Mean temperature in membrane (°C)
W	Channel width (m)
$W_{1}$	Channel width of the inserting carbon-fiber spacers (m)
ω	Absorption efficiency, defined by Equation (23)
$J_{g}$	Mass flux in the gas feed flow side (${\mathrm{mol}\;m}^{-2}s^{-1}$)
$J_{i}$	Mass flux, i=carbon,empty (${\mathrm{mol}\;m}^{-2}s^{-1}$)
$J_{\ell }$	Mass flux in the liquid absorbent flow side (${\mathrm{mol}\;m}^{-2}s^{-1}$)
$J_{m}$	Mass flux in the membrane (${\mathrm{mol}\;m}^{-2}s^{-1}$)
$\omega_{\exp}$	Experimental result of CO_2_ absorption flux (${\mathrm{mol}\;m}^{-2}s^{-1}$)
$\omega_{cal}$	Theoretical predicted CO_2_ absorption flux (${\mathrm{mol}\;m}^{-2}s^{-1}$)
${\left|Y_{m}\right|}_{\ell n}$	Natural log mean CO_2_ mole fraction in the membrane
z	Axial coordinate along the flow direction (m)
Greek letters	
$\alpha^{E}$	Mass transfer enhancement factor
β	Aspect ratio of the channel
$\delta_{m}$	Thickness of membrane (µm)
ε	Membrane porosity
$\bar{\nu }$	Average velocity (${\;m\;s}^{-1}$)
$\rho_{i}$	Density (${\;\mathrm{kg}\;m}^{-3}$),$\;i={\mathrm{CO}}_{2},MEA$
$\gamma_{m}$	Concentration polarization coefficients
Subscripts	
1	Membrane surface on gas side
2(l)	Liquid phase on membrane surface on MEA side
2 ( g)	Gas phase on membrane surface on MEA side
a	In the gas feed flow channel
b	In the liquid absorbent flow channel
cal	Calculated results
carbon	Inserting carbon-fiber as supporters
empty	Inserting nylon fiber as supporters
exp	Experimental results
in	Inlet
out	Outlet
theo	Theoretical predictions
